# A novel marker integrating multiple genetic alterations better predicts platinum sensitivity in ovarian cancer than HRD score

**DOI:** 10.3389/fgene.2023.1240068

**Published:** 2023-09-05

**Authors:** Fan Yang, Wei Wei, Ganghua Li, Qiongyu Lan, Xiwei Liu, Lin Gao, Chao Zhang, Jiangtao Fan, Jundong Li

**Affiliations:** ^1^ Department of Gynecologic Oncology, Sun Yat-sen University Cancer Centre, State Key Laboratory of Oncology in South China, Collaborative Innovation Center for Cancer Medicine, Guangzhou, Guangdong, China; ^2^ GenePlus-Shenzhen, Shenzhen, Guangdong, China; ^3^ Department of Oncology, The Second Affiliated Hospital of Nanchang University, Nanchang, Jiangxi, China; ^4^ Department of Gynecology, The First Affiliated Hospital of Guangxi Medical University, Nanning, Guangxi, China

**Keywords:** Pt-score, platinum sensitivity, platinum-based chemotherapy, HRD score, ovarian cancer, genetic features

## Abstract

**Introduction:** Platinum-based chemotherapy is the first-line treatment strategy for ovarian cancer patients. The dismal prognosis of ovarian cancer was shown to be stringently associated with the heterogeneity of tumor cells in response to this therapy, therefore understanding platinum sensitivity in ovarian cancer would be helpful for improving patients’ quality of life and clinical outcomes. HRDetect, utilized to characterize patients’ homologous recombination repair deficiency, was used to predict patients’ response to platinum-based chemotherapy. However, whether each of the single features contributing to HRD score is associated with platinum sensitivity remains elusive.

**Methods:** We analyzed the whole-exome sequencing data of 196 patients who received platinum-based chemotherapy from the TCGA database. Genetic features were determined individually to see if they could indicate patients’ response to platinum-based chemotherapy and prognosis, then integrated into a Pt-score employing LASSO regression model to assess its predictive performance.

**Results and discussion:** Multiple genetic features, including bi-allelic inactivation of BRCA1/2 genes and genes involved in HR pathway, multiple somatic mutations in genes involved in DNA damage repair (DDR), and previously reported HRD-related features, were found to be stringently associated with platinum sensitivity and improved prognosis. Higher contributions of mutational signature SBS39 or ID6 predicted improved overall survival. Besides, arm-level loss of heterozygosity (LOH) of either chr4p or chr5q predicted significantly better disease-free survival. Notably, some of these features were found independent of HRD. And SBS3, an HRD-related feature, was found irrelevant to platinum sensitivity. Integrated all candidate markers using the LASSO model to yield a Pt-score, which showed better predictive ability compared to HRDetect in determining platinum sensitivity and predicting patients’ prognosis, and this performance was validated in an independent cohort. The outcomes of our study will be instrumental in devising effective strategies for treating ovarian cancer with platinum-based chemotherapy.

## 1 Introduction

Ovarian cancer is a highly prevalent neoplasm, estimated to account for 22,240 new cases and 14,070 deaths in the United States in 2018 (Torre et al., 2018). Noticing the relatively high aggressiveness and low curative rates (Lengyel, 2010), multiple clinical strategies have been developed for disease control and prognosis improvement (Eisenhauer, 2017). Though targeted therapies demonstrated a salutary effect among ovarian cancer patients in advanced stage, a combination of surgery and subsequent platinum (Pt)-based chemotherapy has emerged as standard of care (Cho and Shih, 2009; Jayson et al., 2014). Regarding the inner connection between elevated intra-tumor heterogeneity (ITH) and dismal chemotherapeutic outcomes, multifaceted investigations have been conducted for the derivation of biomarkers possessing power in therapeutic outcome prediction (Yu et al., 2008). Additionally, severe side effects including neurotoxicity and nephrotoxicity commonly cause detrimental effects on patients, so that compromise patients’ benefit and even pose threats to their life (McWhinney et al., 2009; Johnstone et al., 2014). Consequently, the successful identification of sensitive surrogates for the prognostication of Pt-based chemotherapeutic outcomes as well as drug resistance indicators can provide exceptional guidance for treatment decisions and reach an equilibrium between effectiveness and safety.

Quintessentially, Pt-based chemotherapy exert their functions by reacting with the purine bases of DNA, forming inter-strand and/or intra-strand cross-links between nucleotides, hence alter the double helix structure of DNA (Takahara et al., 1995) and impact various biological processes that rely on DNA as their foundation. During the process of transcription, when the DNA repair machinery capitulates in the process of efficacious lesion repair, apoptosis in tumor cells will be reanimated (Johnstone et al., 2014) and impel the accomplishment of disease control. Based on the pharmacodynamical understanding of Pt-based chemotherapy, numerous factors have been demonstrated to be associated with Pt sensitivity and resistance. For instance, the presence of mutations on driver genes including *TP53*, *CCNE1* and *EGFR* both reduce the drug trans-plasma membrane trafficking efficiency and increase the amount of glutathione. Overexpression of metallothionein also closely relates to patients’ resistance to Pt-based chemotherapy (Schilder et al., 1990; Holford et al., 2000), considering the therapeutic sensitivity inherent in dysfunctions of DNA damage repair (Martin et al., 2008; Basourakos et al., 2017). Additionally, the homologous recombination deficiency (HRD) characterized by *BRCA* gene mutation has been observed in various malignancies, including ovarian and breast cancer (Abkevich et al., 2012; Polak et al., 2017; Riaz et al., 2017; Chartron et al., 2019). The widespread of DNA repair mechanism deficiency has become the evidence in predicting patients’ response to cancer drugs, such as poly-(ADP-ribose) polymerase (PARP) inhibitors and Pt-based chemotherapy (McCabe et al., 2006; Mukhopadhyay et al., 2012; Keung et al., 2019; Póti et al., 2019).

Regarding HRD provokes genomic scars and the extent of HRD demonstrated a strong connection with various biological processes, several methods have been proposed for the precise quantification of HRD, which simultaneously exhibited ability for the prediction of patients’ response to Pt-based chemotherapy (Davies et al., 2017; Staaf et al., 2019). The HRD score, defined as the unweighted sum of several signatures of genomic instability including loss of heterozygosity (LOH), telomeric allelic imbalance (TAI) and large-scale transitions (LST), has drawn growing attention because of its high accuracy and strong clinical relevance (Rempel et al., 2022). However, the connection between HRD score and Pt-based therapeutic sensitivity in ovarian cancer needs further study, and the integration of HRD score with specific genetic alterations might improve the predictive performance. Therefore, we hypothesized that the integration of HRD related signatures and other genomic alterations may promote the development of a method with high sensitivity and specificity in predicting ovarian cancer patients’ response to Pt-based chemotherapy.

Here, we used whole-exome sequencing (WES) data of 196 ovarian cancer patients from the TCGA database to identify the genetic features between Pt-sensitive patients and Pt-resistant patients. And then, we established a novel model by integrating DNA damage repair (DDR) deficiency and other genetic features, which substantially increased the sensitivity and specificity in predicting patients’ response to Pt-based chemotherapy compared to HRD score, so that it could facilitate more precise drug regimen designing and reduce risks for potential side effects.

## 2 Materials and Methods

### 2.1 Patient selection and original data accessing

WES data of 578 ovarian cancer patients were obtained from the TCGA database (accessed in August 2022). To study the relationship of DDR deficiency and Pt sensitivity, patients who underwent Pt-based chemotherapy and with 1) detailed clinical information on Pt sensitivity (either sensitive or resistant) and response to Pt-based chemotherapy (based on RECIST 1.1 criteria); 2) bi-allelic alteration status in HRD genes characterized previously in a publication (Riaz et al., 2017) were included in our study. Eventually, a total of 196 ovarian cancer patients from the TCGA database were enrolled in this study. Detailed clinicopathological information of all included patients was listed in Supplementary Table S1, and these samples were collected prior to any treatment. We used the WES data and clinicopathological information of 196 ovarian cancer patients from the TCGA database to construct the Pt-score subsequently. The validation cohort which including 60 ovarian cancer patients with WES data, was downloaded from public database (CNGBdb accession: CNP0001937).

### 2.2 The derivation of Pt sensitivity associated genetic alterations

Bi-allelic alterations in HR genes considering germline mutations and small fragment insertions and deletions (Indels), somatic mutations, and loss of heterozygosity (LOH) were obtained from a previous publication (Riaz et al., 2017). To be more precise, four types of bi-allelic inactivation including a) germline pathogenic mutations with LOH; b) germline pathogenic mutations with heterozygous somatic pathogenic mutations; c) somatic pathogenic mutations with LOH; d) somatic pathogenic mutations with heterozygous somatic pathogenic mutations were included in our study. Additionally, somatic single nucleotide variations (SNVs) and Indels of ovarian cancer patients were collected from the TCGA database. Variants with high frequency among unrelated individuals were firstly filtered out as previously reported (Li et al., 2017), then further filtered in a way similar to the method demonstrated in previous research (Angus et al., 2019). More specifically, alternations including nonsense mutations, missense mutations, mutations on splice sites and in frame/frameshift indels were initially collected. As for missense mutations, only those matching two or more of the following *in silico* functional criteria: 1) prediction score of 0–0.05 with SIFT (Sorting Intolerant from Tolerant) method; 2) “possibly damaging” or “probably damaging” in Polymorphism Phenotyping-2 (Polyphen2) algorithm or 3) reported in COSMIC database with a FATHMM-MKL (Functional Analysis through Hidden Markov Models) score larger than 0.5 (accessed in August 2020) were retained. We also quantified mutational signatures for the derivation of more comprehensive information. Trinucleotide altering patterns of unfiltered somatic SNVs and fragment changing patterns of unfiltered Indels in our cohort were matched to the signatures described in the COSMIC database using YAPSA software (version 3.11). Exposing contribution of each signature in samples was quantified and only signatures contributing to more than 1% alterations were illustrated and considered in the subsequent analyses.

Apart from mutational alternations, arm-level copy number loss was simultaneously collected in our comparative analyses due to the lack of studies related to the role of arm-level LOH in Pt sensitivity prediction. As for the detection of arm-level copy number loss, fragment-level copy number information from ABSOLUTE in TCGA database was obtained and only loss covering at least 90% of chromosome arm length were collected. We defined four types of arm-level LOH: 1) both copy numbers on major and minor allele equaled to 0; 2) loss of heterozygosity (LOH) when major and minor allele copy numbers equaled to 1 and 0 respectively; 3) copy neutral LOH (CNLOH) when major and minor allele copy numbers equaled to 2 and 0 respectively; 4) LOH at high ploidy when minor allele copy number equaled to 0 whereas major allele copy number was larger than 2. These LOH characteristics and mutational features were gathered for further confirming the prioritization of Pt sensitivity associated genetic features.

### 2.3 Construction of Pt sensitivity prediction model

HRD bi-allelic alterations, somatic mutations, arm-level LOH, and other genetic features were respectively compared between patients stratified by Pt sensitivity. To reduce possible heterogeneity, we only selected genes with the highest mutational frequency together with mutations in well-characterized genes involved in DDR pathway (Knijnenburg et al., 2018). A catalogue containing all analyzed DDR genes was listed in Supplementary Table S2. Only the features showing significantly different proportion between two groups and demonstrated high correlation with patients’ survival were selected as candidates for further model construction. The candidate features were eventually integrated as Pt score employing the LASSO regression model. The predictive performance of the LASSO-Pt score was trained on the TCGA-sourced data and verified in an external dataset, and then compared with the HRDscore derived by the HRDetect method.

### 2.4 Statistical methods

Two-sided Mann-Whitney and Fisher’s exact tests were performed on GraphPad Prism (version 7.01) or R (version 3.6.1) to quantify distribution differences. Log-rank tests were employed to compare the Kaplan-Meier survival curves of ovarian cancer patients on GraphPad Prism (version 7.01). LASSO model and the corresponding AUC curves were generated using R software (version 3.6.1). The predictive performance of LASSO-Pt score and HRDscore was compared by DeLong’s test. Statistical significance was defined as *p* < 0.05 for the statistical analyses.

## 3 Results

### 3.1 Specific genetic alterations were observed in patients with distinct response to Pt-based chemotherapy

**FIGURE 1:**
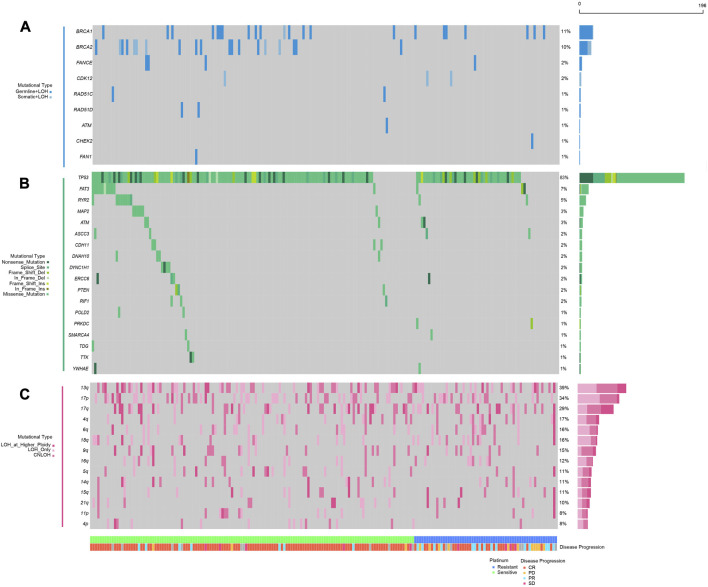
Mutational landscape of homologous repair and DNA damage repair-related genes in platinum sensitive or resistant ovarian cancer patients; **(A)**, double hit (including germline SNVs, somatic SNVs, and loss of heterogeneity) mutations in HR-related genes (*BRCA1*, *BRCA2*, *FANCE*, *CDK12*, *RAD51C*, *RAD51D*, *ATM*, *CHEK2*, *FAN1*); **(B)** somatic single nucleotide variations and small insertions and deletions in genes involved in DNA damage repair (*TP53*, *FAT3*, *RYR2*, *MAP2*, *ATM*, *ASCC3*, *CDH11*, *DNAH10*, *DYNC1H1*, *ERCC6*, *PTEN*, *RIF1*, *POLD2*, *PRKDC*, *SMARCA4*, *TDG*, *TTK*, *YWHAE*); **(C)** loss of heterogeneity in specific chromosomal arms; the panel at the bottom shows classification of patients regarding platinum resistance, patients’ response to platinum chemotherapies.

Regarding the recent revitalization of demand in Pt sensitivity prediction for ovarian cancer, several factors including homologous recombination status, specific somatic mutations and ITH demonstrated association with Pt sensitivity, albeit the limited specificity (Luo et al., 2020). Given that bi-allelic inactivation might contribute to the loss of functions in certain genes and provide unprecedented information, we obtained the bi-allelic mutational data of HR-related genes in 196 ovarian cancer patients from TCGA database and explored their association with Pt sensitivity. As shown in Figure 1A, only germline pathogenic mutations with LOH and somatic pathogenic mutations with LOH were identified in HR-related genes in the curated cohort. Genes including *BRCA1* (22/196, 11.2%) and *BRCA2* (20/196, 10.2%) exhibited the highest bi-allelic mutational frequency, in line with the reported findings (Riaz et al., 2017).

As previously stated, considering the theoretical associations between DDR mechanism and Pt-based chemotherapy response, we further accessed all the somatic SNVs in these 196 patients from TCGA database and scrutinized the alterations in all DDR-related genes collected from a previous study (details in Materials and Methods). Recurrently mutated driver genes were also encompassed in our research, given that they were indicated to play essential roles in cell survival and Pt sensitivity. After filtration, *TP53* (163/196, 83.2%), *FAT3* (14/196, 7.1%), *RYR2* (10/196, 5.1%) and *MAP2* (6/196, 3.1%) demonstrated highest mutational frequency (Figure 1B). Besides, sporadic alternations were observed among DDR-related genes including *ERCC6* (4/196, 2.0%), *PTEN* (3/196, 1.5%) and *RIF1* (3/196, 1.5%), most of which showed an enrichment in Pt-sensitive patients.

Analyses on copy number variation (CNV) have provided an insightful mechanism to decipher critical conundrums for Pt sensitivity in ovarian cancer. The connection between long-segment copy number loss and HRD endorsed its application in Pt therapeutic sensitivity prediction. However, arm-level loss was habitually disregarded since chromosome arm aneuploidy was often caused by mechanisms other than HRD. To assess whether loss of copy number at arm level was associated with patients’ response to Pt-based chemotherapy, the CNV data of these 196 patients were also accessed through TCGA database. The somatic copy number alteration (SCNA) was called using ABSOLUTE and any loss of copy number at the arm-level was exhibited in Figure 1C. These results showed that chromosomal arm-level loss was common among patients, including 13q (76/196, 38.8%), 17p (67/196, 34.2%), 17q (57/196, 29.1%), 4q (33/196, 16.8), 6q (31/196, 15.8%), and others. At the same time, these changes had been considered in the subsequent analyses to explore their potential correlations with Pt sensitivity (Figure 1C).

### 3.2 Identification of specific biomarker to associate with Pt sensitivity

To study whether these genetic alterations (Figure 1) are associated with Pt sensitivity, the information of patients’ response to Pt-based chemotherapy and disease progression were assessed. The result showed that a significantly higher proportion of patients with *BRCA1/2* biallelic inactivation (85.4%) was observed to be sensitive to Pt-based chemotherapy compared to wild-type *BRCA1/2* patients (65.2%) (*p* = 0.013) (Table 1). Similarly, a significantly higher proportion of patients with bi-allelic inactivation in HR-related genes (81.1%), somatic mutations in DDR pathways (100%), somatic mutations in genes involved in microtubule functions (100%) or arm-level copy number loss in chromosome 4p (93.8%) was observed sensitive to Pt-based chemotherapy compared to their counterparts (65.0%, 67.4%, 67.0% and 67.2% respectively) (*p* = 0.036, 0.019, 0.006, and 0.025 respectively) (Table 1). Patients with arm-level copy number loss in either chromosome 5q or 4p were also found significantly enriched in the Pt-sensitive group, with a lower *p*-value (0.003). However, other frequently mutated genes or pathways, including somatic mutations in *TP53*, *FAT3*, *RYR2*, pathways engaged in calcium channel activities, and arm-level copy number loss in 13q, 17p, 17q and others, were not found associated with patients’ sensitivity to Pt-based chemotherapy. No significant association could be observed between any genetic alterations and patients’ pathological response as well, which was defined as whether patients achieved complete response or partial response, but borderline significance was found between copy number loss of either chromosome 5q or 4p and patients’ pathological response (*p* = 0.084) (Table 1).

**TABLE 1:** Multiple genetic changes were stringently associated with platinum sensitivity but not associated with clinicopathological characteristics. Some factors are independent of BRCA mutations, indicating they are irrelevant to HRD.

Event	BRCA1/2-double hit			HR-double hit			DDR-somatic			Microtubule-related genes			Chr4p-Loss			Chr5q/4p-Loss		
Status	Mutated (*n* = 41)	Wild-type\(*n* = 155)	*p*	Mutated\(*n* = 53)	Wild-type (*n* = 143)	*p*	Multiple mutations (*n* = 12)	1 or no mutation (*n* = 184)	*p*	Mutated (*n* = 14)	Wild-type (*n* = 182)	*p*	Yes (*n* = 16)	No (*n* = 180)	*p*	Yes (*n* = 37)	No (n = 159)	*p*
Age at diagnosis (years)																		
Median	50	60	-	51	60.5	-	66	58	-	61	58	-	56	59	-	59	57.5	-
Range	38–78	38–87		38–78	38–87		40–73	38–87		40–81	38–87		40–81	38–87		40–81	38–87	
Histological Grade, n (%)																		
G1-G2	9 (22.0)	18 (11.6)	0.117	10 (18.9)	17 (11.9)	0.143	1 (8.3)	26 (14.1)	1	2 (14.3)	25 (13.7)	1	1 (6.3)	26 (14.4)	0.697	4 (10.8)	23 (14.5)	0.787
G3-G4	22 (53.7)	97 (62.6)		27 (50.9)	92 (64.3)		6 (50.0)	113 (61.4)		11 (78.6)	108 (59.3)		11 (68.8)	108 (60.0)		24 (64.9)	95 (59.7)	
Unknown	10 (24.4)	40 (25.8)		16 (30.2)	34 (23.8)		5 (41.7)	45 (24.5)		1 (7.1)	49 (26.9)		4 (25.0)	46 (25.6)		9 (24.3)	41 (25.8)	
Pt Sensitivity, n (%)																		
Sensitive	35 (85.4)	101 (65.2)	0.013 (*)	43 (81.1)	93 (65.0)	0.036 (*)	12 (100)	124 (67.4)	0.019 (*)	14 (100)	122 (67.0)	0.006 (**)	15 (93.8)	121 (67.2)	0.025 (*)	33 (89.2)	103 (64.8)	0.003 (**)
Resistant	6 (14.6)	54 (34.8)		10 (18.9)	50 (35.0)		0	60 (32.6)		0	60 (33.0)		1 (6.3)	59 (32.8)		4 (10.8)	56 (35.2)	
Pathological Response, n (%)																		
Response (CR + PR)	37 (90.2)	122 (78.7)	0.419	48 (90.6)	111 (77.6)	0.322	11 (91.7)	148 (80.4)	0.364	14 (100)	145 (79.7)	0.222	13 (81.3)	146 (81.1)	0.377	31 (83.8)	128 (80.5)	0.084
No	3 (7.3)	20 (12.9)		4 (7.5)	19 (13.3)		0	23 (12.5)		0	23 (12.6)		0	23 (12.8)		1 (2.7)	22 (13.8)	
Unknown	1 (2.4)	13 (8.4)		1 (1.9)	13 (9.1)		1 (8.3)	13 (7.1)		0	14 (7.7)		3 (18.8)	11 (6.1)		5 (13.5)	9 (5.7)	
BRCA1/2-Double Hit																		
Mutated	—	—	—	—	—	—	4 (33.3)	37 (20.1)	0.28	3 (21.4)	38 (20.9)	1	6 (37.5)	35 (19.4)	0.108	8 (21.6)	33 (20.8)	1
Wild-Type	—	—		—	—		8 (66.7)	147 (79.9)		11 (78.6)	144 (79.1)		10 (62.5)	145 (80.6)		29 (78.4)	126 (79.2)	

The bold values provided in Table mean that there is a statistically significant difference (*p* < 0.05) between the two groups.

Considering certain mutations might be enriched in patients with specific clinicopathological status, we also looked at the association between these genetic alterations and clinicopathological features such as patients’ age at diagnosis and tumor histological grades. As a result, no significant association could be observed between genetic alterations and clinicopathological features (Table 1). Given that *BRCA1/2* were well-accepted biomarkers to indicate HRD to date, we analyzed the association between these genetic alterations and *BRCA1/2* bi-allelic inactivation, trying to see whether these genetic alterations were related to HRD. The result showed that all genetic alterations which showed a strong association with Pt sensitivity were uncorrelated with *BRCA1/2* mutations, indicating that these alterations were biomarkers in predicting patients’ response to Pt sensitivity and independent to HRD (Table 1).

**FIGURE 2:**
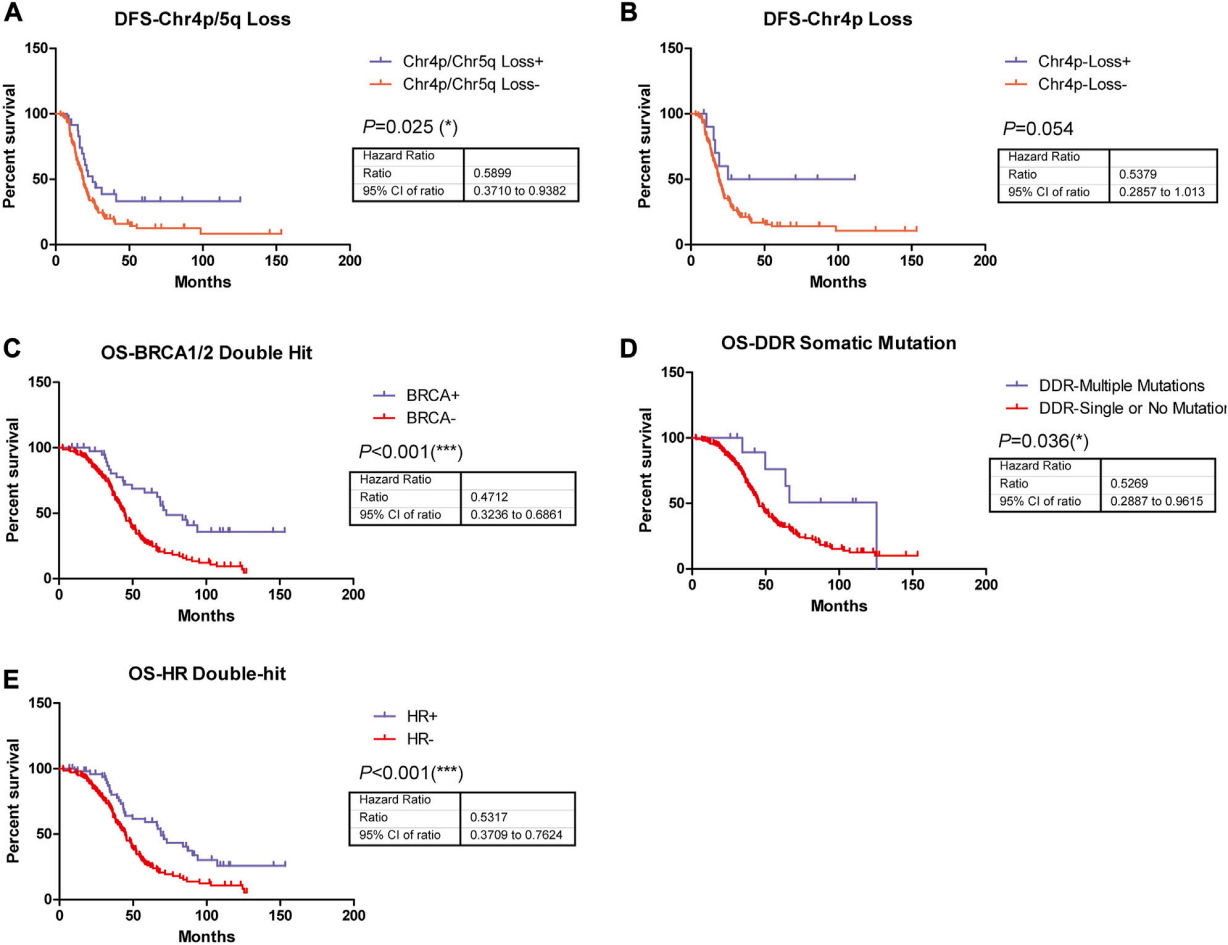
Among these genetic alterations, five were observed to be able to significantly predict patients’ disease-free survival or overall survival. **(A)** Copy number loss of chromosome 4p/5q indicated significantly improved DFS (*p* = 0.025, HR = 0.5899, 95% CI, ranged from 0.3710 to 0.9382) in ovarian patients who received Pt-based chemotherapies. **(B)** Copy number loss of chromosome 4p indicated significantly improved DFS (*p* = 0.054, HR = 0.5379, 95% CI, ranged from 0.2857 to 1.013) in ovarian patients who received Pt-based chemotherapies. **(C)** Patients harboring BRCA1/2 biallelic inactivation was associated with significantly better OS (*p* < 0.001, HR = 0.4712, 95% CI, ranged from 0.3236 to 0.6861) in ovarian patients who received Pt-based chemotherapies. **(D)** More than 1 somatic mutation in DDR-related genes was associated with significantly better OS (*p* = 0.036, HR = 0.5269, 95% CI, ranged from 0.2887 to 0.9615) in ovarian patients who received Pt-based chemotherapies. **(E)** Biallelic inactivation in HR pathway was associated with significantly better OS (*p* < 0.001, HR = 0.5317, 95% CI, ranged from 0.3709 to 0.7624) in ovarian patients who received Pt-based chemotherapies.

In addition, the survival analysis demonstrated that copy number loss of chromosome 4p/5q indicated significantly improved disease-free survival (DFS) in ovarian cancer patients who received Pt-based chemotherapy (Figure 2A), suggesting these copy number alterations might play roles in disease short-term progression, recurrence, or metastasis, while no significant difference could be observed in DFS of patients with copy number loss of chromosome 4p compared to their wild type counterparts (Figure 2B). Nonetheless, patients harboring *BRCA1/2* biallelic inactivation, biallelic inactivation in the HR pathway, or more than one somatic mutation in DDR-related genes exhibited longer overall survival (OS) time in comparison with their counterparts (Figures 2C–E), indicating their associations with better prognosis in ovarian cancer patients who received Pt-based chemotherapy.

### 3.3 Mutational signatures predict Pt sensitivity and prognosis

**FIGURE 3:**
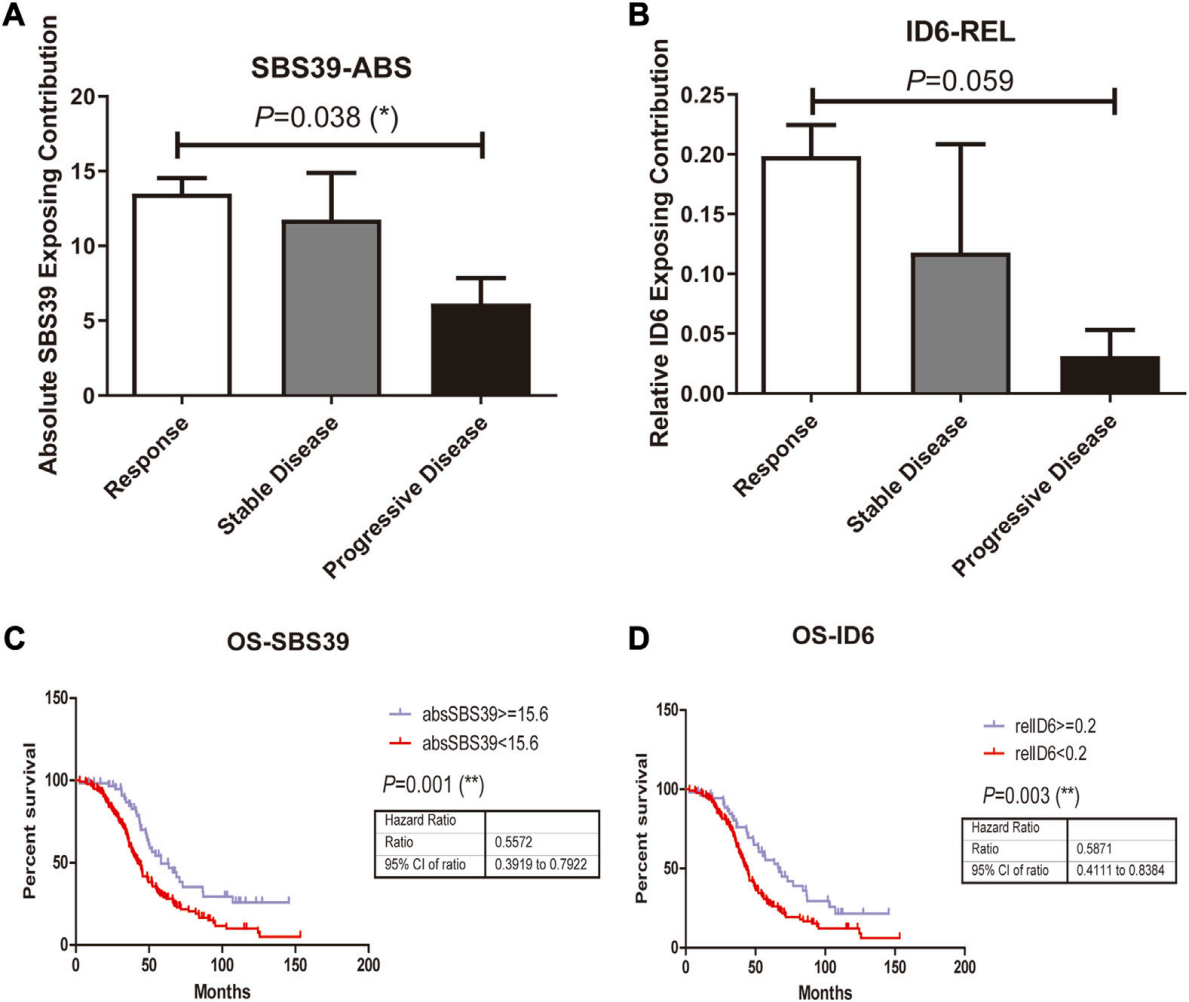
Mutations in platinum resistant and sensitive ovarian cancer patients were attributed to PCAWG reported SBS and Indel signatures, where the difference was observed in attributing signature scores between responding group and the resistant group. **A-B**, SBS39-abs and ID6-rel Exposing Contribution scores among patients with different therapeutic outcomes. **C**, High SBS39-abs score was associated with significantly improved OS (*p* = 0.001, HR = 0.5672, 95% CI, ranged from 0.3919 to 0.7922) in ovarian patients who received Pt-based chemotherapies. **D**, High ID6-rel score was associated with significantly better OS (*p* = 0.003, HR = 0.5871, 95% CI, ranged from 0.4111 to 0.8384) in ovarian patients who received Pt-based chemotherapies.

**FIGURE 4:**
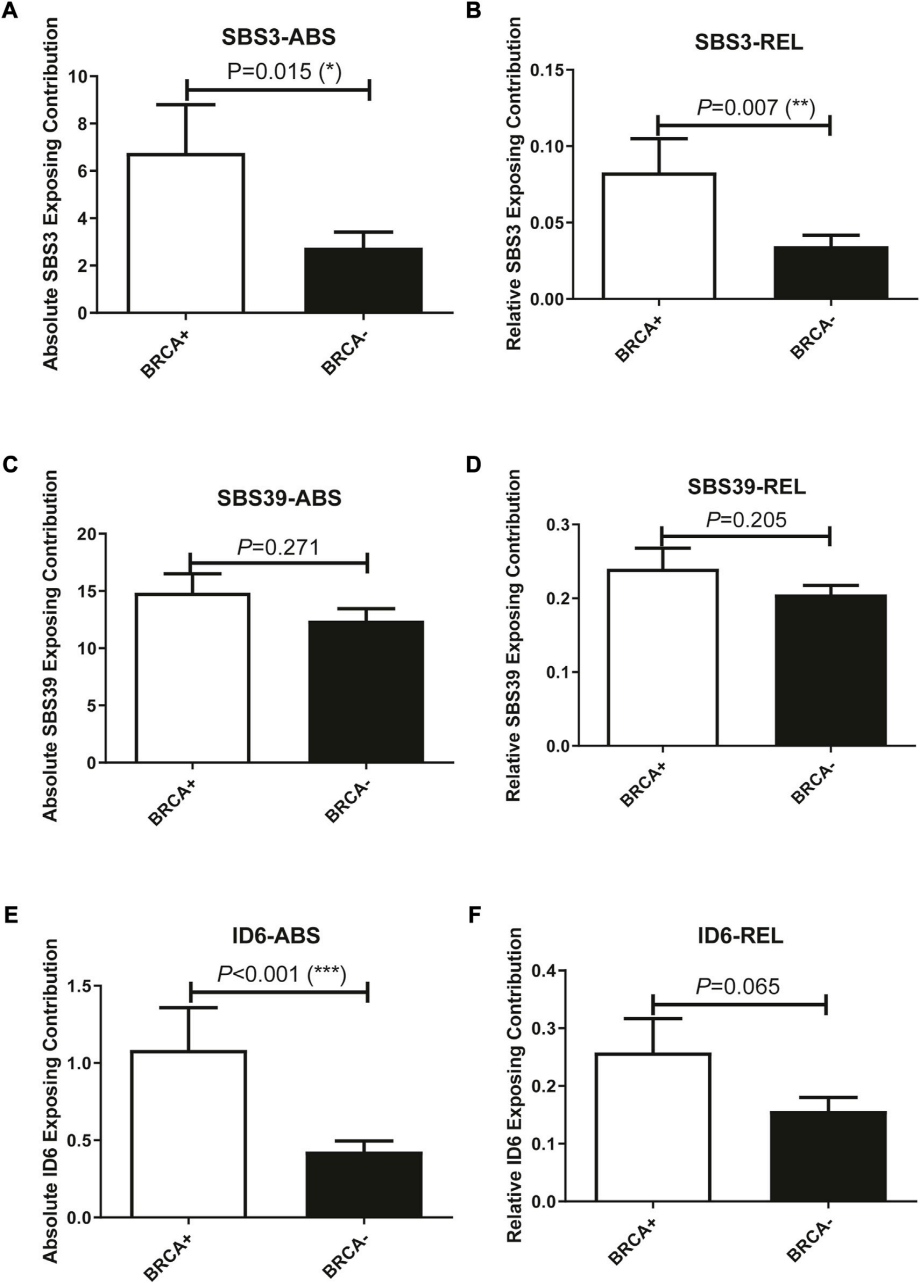
Exposing scores of SBS3 and ID6 were significantly associated with *BRCA* mutations, indicating the association between these two signatures with HRD, while SBS39 was independent of HRD. **(A, B)**, SBS3-ABS, and SBS3-REL were significantly associated with BRCA mutations. (One-way ANOVA followed by Tukeys *post hoc* test). **(C, D)**, SBS39-ABS, and SBS39-REL were insignificantly associated with BRCA mutations. (One-way ANOVA followed by Tukeys *post hoc* test). **(E, F)**, ID6-ABS, and ID6-REL were significantly associated with BRCA mutations. (One-way ANOVA followed by Tukeys *post hoc* test).

In addition to studying whether the genetic alterations are associated with Pt sensitivity in ovarian cancer patients, we also looked at the association between mutational signature and patients’ disease progression. The exposing score of each SBS signature was calculated by YAPSA software. A significant difference was observed in the absolute SBS39 exposing score and the relative ID6 exposing score between the responding group and the non-responding group, both showed higher exposing contribution in the responding group (*p* = 0.038 and 0.059 for absolute SBS39 exposing score and relative ID6 exposing score, Figures 3A, B). Survival analysis demonstrated that patients with higher absolute SBS39 or relative ID6 exposing scores showed significantly improved OS (*p* = 0.001 and 0.003 respectively) (Figures 3C,D). Suggesting SBS39 and ID6 might play important roles in long-term survival. Previously, a high SBS3 exposing score was shown to be one of the genetic features in patients with HRD. However, in our result, no significant difference could be observed in SBS3 exposing scores between the responding group and the non-responding group. Furthermore, we also analyzed whether the exposing scores of these SBS were associated with HRD in our cohort. The result showed that SBS3 and ID6 were significantly associated with *BRCA* mutations, indicating the correlations between these two mutational signatures and HRD, while SBS39 was observed independent of HRD (Figure 4).

### 3.4 HRD-associated whole-genome alterations predict patients’ Pt sensitivity and prognosis

**FIGURE 5:**
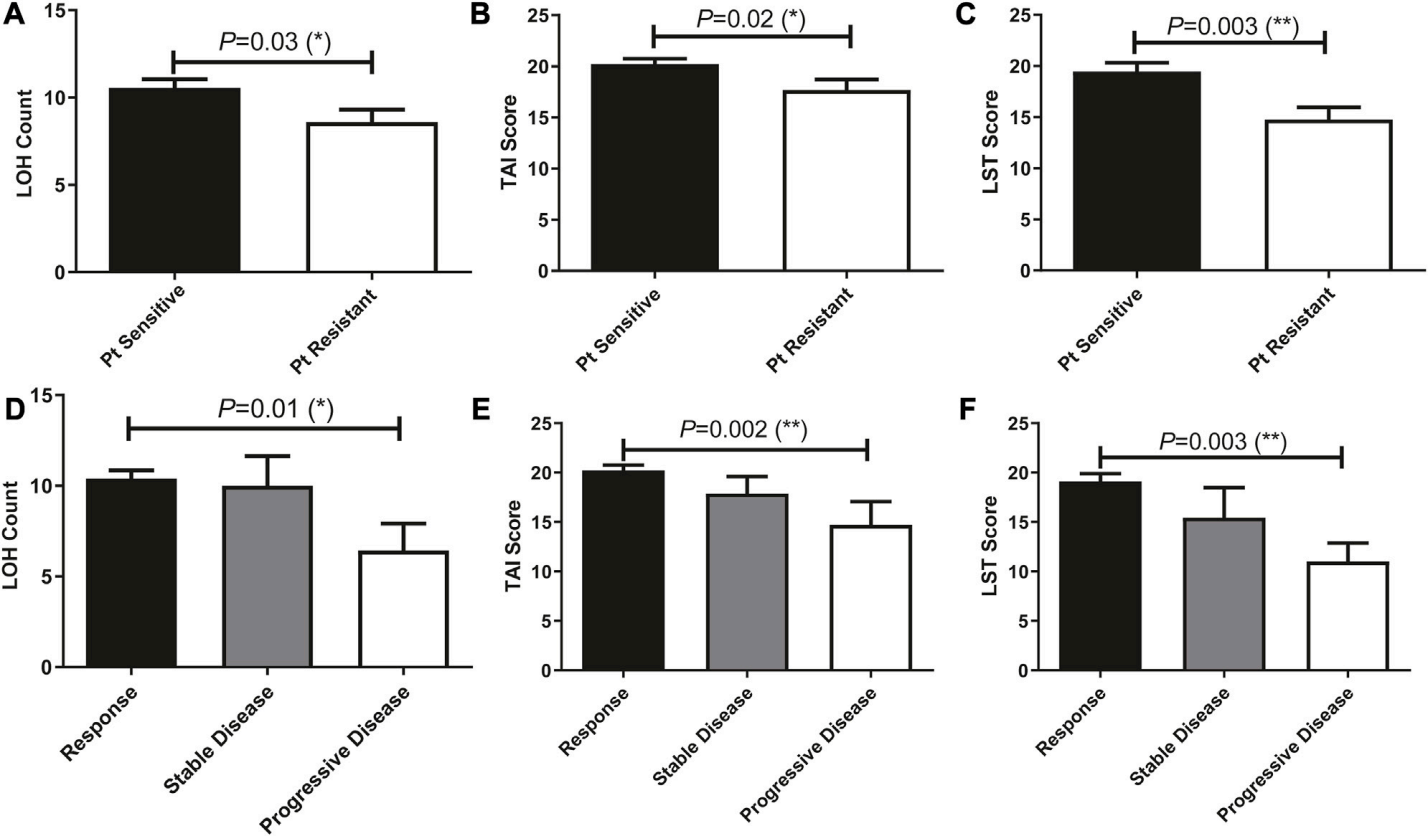
Prevalence of HRD-associated whole-genome alterations (stringently associated with BRCA mutations) were significantly different among patients with different therapeutic outcomes and can predict patients’ response to Pt treatment. **(A–C)**, HRD-associated whole-genome alterations were significantly associated with Pt sensitivity. **(D–F)**, HRD-associated whole-genome alterations were significantly associated with patients’ disease progress.

TAI, LST, and LOH scores of patients who treated with Pt-based chemotherapy were previously reported to be stringently associated with HRD and predictive of disease progression. In our cohort, these genetic features that contributed to the HRD score were significantly associated with patients’ sensitivity to Pt-based chemotherapy and disease progression (Figure 5). We then analyzed the ability of these scores in predicting patients’ sensitivity and disease progression after receiving Pt-based chemotherapy in all 196 patients.

### 3.5 The integrating model showed improved predictive ability to predict Pt sensitivity and patients’ prognosis compared to the HRDetect method

**FIGURE 6:**
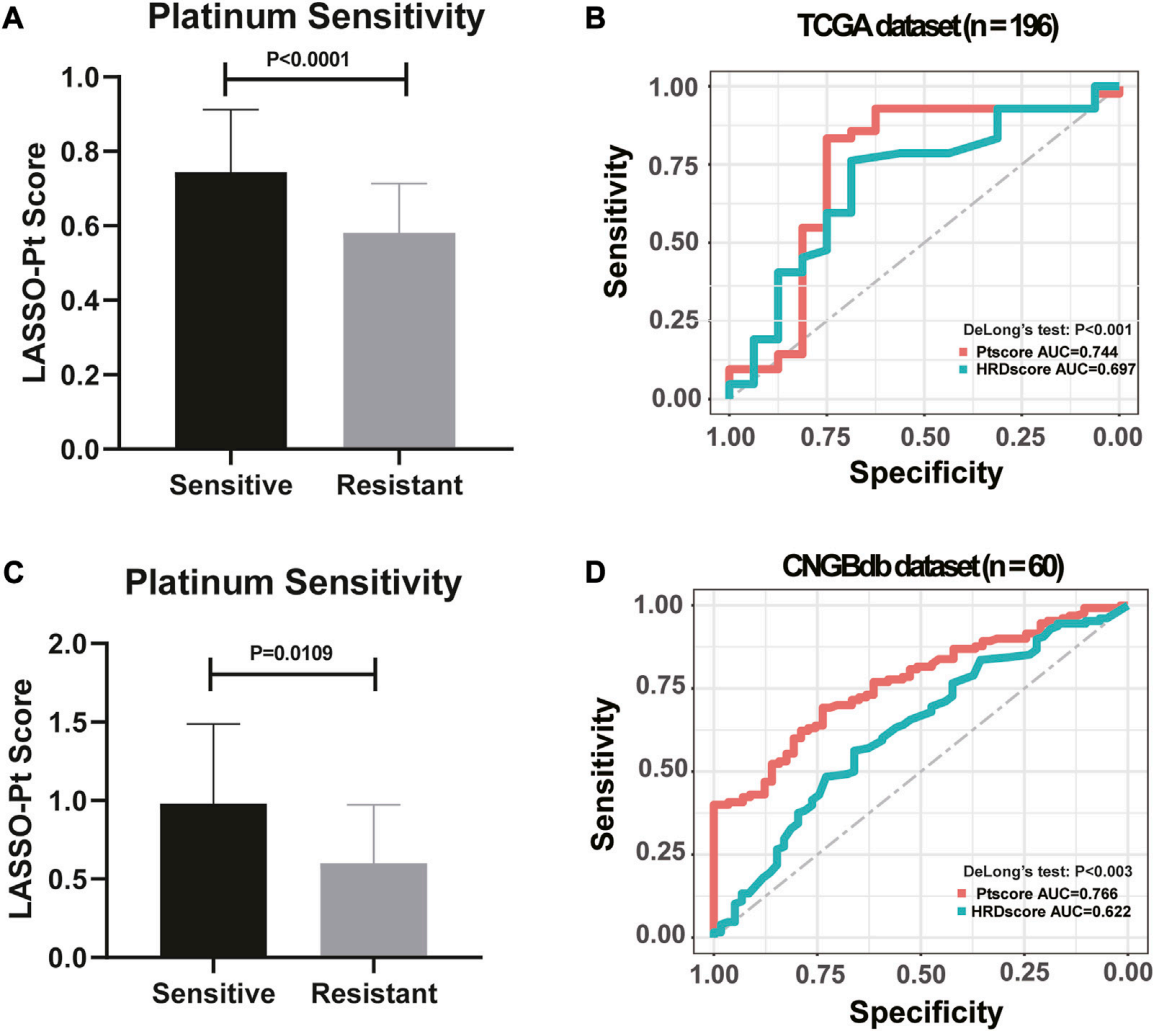
LASSO model integrating genetic markers showed an improved predictive ability to indicate platinum resistance (DeLong’s Test, *p* < 0.001). **(A)** High LASSO-Pt scores were significantly associated with Platinum Sensitivity in TCGA dataset. (One-way ANOVA followed by Tukeys *post hoc* test). **(B)** Comparison of HRD scores and LASSO-Pt scores were based on DeLong’s Test in predicting patients’ sensitivity to Pt chemotherapy in TCGA dataset. **(C)** High LASSO-Pt scores were significantly associated with platinum Sensitivity in CNGBdb dataset. (One-way ANOVA followed by Tukeys *post hoc* test). **(D)** Comparison of HRD scores and LASSO-Pt scores were based on DeLong’s Test in predicting patients’ sensitivity to Pt chemotherapy in CNGBdb dataset.

HRD bi-allelic alterations, somatic mutations in DDR pathways, arm-level LOH, mutational signatures and other genetic features that showing significantly different prevalence between the Pt responding group and the non-responding group were screened out to establish a biomarker for Pt sensitivity. Notably, patients who were sensitive to Pt-based chemotherapy were observed with significantly higher LASSO-Pt score than patients who were resistant to Pt-based chemotherapy in both TCGA dataset (Figure 6A, *p* < 0.0001) and CNGBdb dataset (Figure 6C, *p* = 0.0109).

Genetic features were integrated into an Pt score employing the LASSO regression model. The predictive ability of the LASSO-Pt score was then further validated in all 196 ovarian cancer patients. Previously, HRD score gauged using HRDetect method was shown with satisfactory predictive ability of Pt sensitivity in ovarian cancer patients (Sztupinszki et al., 2021). We then compared the performance of the LASSO-Pt score against the HRD score in predicting Pt sensitivity in our cohort. The result showed that the LASSO-Pt score was observed with significantly improved sensitivity and specificity than the HRD score (DeLong’s Test, *p* < 0.001, Figure 6B).

To validate the reliability of LASSO-Pt score, a public (CNGBdb accession: CNP0001937) dataset of 60 ovarian cancer patients with Pt treatment information was downloaded. After training, an AUC value of 0.744 was obtained with CNGBdb dataset, suggesting potential predictive value of these variations in clinical setting (DeLong’s Test, *p* < 0.003, Figure 6D).

## 4 Discussion

The dismal prognosis of ovarian cancer has been found to be strictly associated with the heterogeneity of tumor cells in response to Pt-based chemotherapy. A lot of studies have attempted to investigate the biomarkers associated with platinum sensitivity. For example, high *FZD10*, *MKX*, *FAM83A* and *MYO18B* methylation were related with response to platinum-based chemotherapy in advanced stage high-grade serous ovarian cancer (HGSOC), and these markers might be used to predict platinum sensitivity in HGSOC patients (Tomar et al., 2017). And HRDetect, used to characterize patients’ DNA homologous recombination (HR) deficiency, has been used to predict tumor response to Pt-based chemotherapy due to its great predictive performance (Zhao et al., 2017). However, it is unclear whether each feature that contributes to HRD score is associated with Pt sensitivity and the relevance between high methylation of above four gene markers and Pt sensitivity has to be further validated. In addition, since homologous recombination is only one of the DNA repair mechanisms, biomarkers based solely on HRD may simply possess modest sensitivity and specificity in the prediction of Pt sensitivity. Herein we analyzed the WES data of 196 patients who received Pt-based chemotherapy from the TCGA database to explore the genetic features that might be associated with patients’ response to Pt-based chemotherapy and establish a LASSO model to integrate all associated biomarkers and predict patients’ potential benefit from Pt-based chemotherapy.

Our findings have notable implications for the analysis of DDR consisting of HR and other DNA damage repair mechanism-related genes. When pathogenic variants of *TP53*, *FAT3*, *RYR2*, and somatic variants of *MAP2*, *FAT3*, *RYR2*, *CDH11*, *MAP2*, *DNAH10*, *DYNC1H1*, and so forth, is found in breast or ovarian cancer, it is inferred to be the putative driver mutations of carcinogenesis. However, among 163 carriers of pathogenic somatic *TP53* variants in our study whose tumor LOH status was discernible, we identified that some tumors exhibited loss of DDR-related genes, such as *ERCC6*, *PTEN*, *RIF1*, *POLD2*, *PRKDA*, *SMARCA4*, *TDG*, *TTK* and *VWHAE*. In these cases, though somatic mutations in HR pathway might not be detected, the aberrations of DDR pathway might facilitate tumor responding to Pt-based chemotherapy. Consequently, integration of driver genes with the highest mutation prevalence and DDR-related genes may affect the selection of therapeutic regimen.

Moreover, genetic features previously reported to be associated with HRD were analyzed individually to determine their possible roles in Pt sensitivity, then the features showing significant associations were integrated into a Pt-score employing the LASSO regression model. The results showed that multiple genetic features were significantly associated with Pt sensitivity and indicated better prognosis in patients, including bi-allelic inactivation of *BRCA1/2* genes and the genes in HR pathway, multiple somatic mutations in genes involved in DNA damage repair, and previously reported HRD-related features. And high contribution score of signatures SBS39 or ID6 predicted improved overall survival. Besides, arm-level loss of heterozygosity (LOH) of either chr4p or chr5q predicted significantly better disease-free survival. Notably, some of these features were found independent of HRD, and SBS3, an HRD-related feature, was found irrelevant to Pt sensitivity.

Integrated all candidate biomarkers using the LASSO regression model to yield a LASSO-Pt score, which showed significantly better predictive ability compared to the HRD score in determining Pt sensitivity (*p* < 0.001) and predicting patients’ prognosis (*p* < 0.001, HR = 0.51). Taken together, HRD is, but not the only factor associated with Pt sensitivity, and not all individual features of HRD can predict Pt sensitivity. We established a novel model to determine the LASSO-Pt score of ovarian cancer patients, which substantially increased the sensitivity and specificity in predicting patients’ Pt-sensitivity, therefore contributing to facilitate designing more precise therapy regimen and reduce risks for potential side effects. Nevertheless, as sequencing costs fall, WES provides unique opportunity to integrate diverse biomarkers of genomic instability and mutagenesis within a single protocol.

While the performance of the LASSO-Pt score was promising, algorithmic development had also been envisaged, including the identification of tumors that have developed treatment-resistant alleles. Historic scars of homologous recombination deficiency within genetic changes will be present in a treatment-resistant tumor, possibly leading to a high LASSO-Pt score. However, exploring ongoing historic mutational signatures of homologous recombination deficiency within genetic changes is already a possibility, given the advances in exploiting the digital nature of sequencing technologies to construct phylogenetic trees of each cancer patient (Alexandrov et al., 2013; Yates et al., 2015).

Intratumor heterogeneity has been extensively studied as a confounding factor for obtaining accurate diagnostic measurements from a single biopsy (McGranahan and Swanton, 2017; Marusyk et al., 2020). Previous studies have shown that the HRD score was independent of intratumor heterogeneity in breast cancer (von Wahlde et al., 2017). Here in our study, one of the limitations was we did not analyze the association of LASSO-Pt score with intratumor heterogeneity. However, biopsies at different time points from different anatomical sites of the same patient often produce conflicting HR deficiency status, which requires further studies and a careful evaluation of the use of Pt-based chemotherapy in ovarian cancer patients. At last, modest sample size and the lack of real-world sequencing data might compromise the accuracy and limit the clinical translation of LASSO-Pt score, which awaits further investigations.

Understanding platinum sensitivity and its clinical implications can pose challenges for clinicians. Clinicians can utilize this information to personalize treatment decisions and improve patient outcomes. One potential application of LASSO-Pt score is in treatment planning before starting platinum-based chemotherapy. By analyzing a patient’s genetic profile, clinicians can identify those who are more likely to respond to platinum therapy. This can help guide treatment decisions and optimize patient care. Additionally, LASSO-Pt score can also be utilized during treatment monitoring. Regular assessment of the patient’s genetic markers during chemotherapy can provide valuable information on treatment response. Overall, the inclusion of genetic markers in the treatment paradigm allows for a more personalized and precise approach to ovarian cancer treatment. This can lead to improved treatment outcomes and better quality of life for patients.

## 5 Conclusions

In this study, genetic alterations including copy number variations and mutations in HRD and DDR pathways were associated with clinical benefit of Pt-based chemotherapy in ovarian cancer. Specifically, utilizing WES data from 196 ovarian cancer patients from the TCGA database, we compared the genetic features between Pt sensitive patients and Pt resistant patients, and developed a novel model integrating DNA damage repair (DDR) deficiency and other Pt sensitive genetic features, which substantially increased the sensitivity and specificity in predicting ovarian cancer patients’ response to Pt-based chemotherapy. Our findings uncovered the genetic features that might be independent of HRD but associated with Pt sensitivity and provided a novel approach to integrate multiple features to predict ovarian cancer patients’ response to Pt-based chemotherapy, offering promising evidence for making clinical decisions to ovarian cancer patients.

## Data Availability

The datasets presented in this study can be found in online repositories, and the names of the repository/ repositories and accession number(s) can be found below: TCGA database (https://portal.gdc.cancer.gov/) (accessed in August 2022) and China National GeneBank DataBase (accession: CNP0001937). Besides, the original contributions presented in the study are included in the article/Supplementary Material, further inquiries can be directed to the corresponding authors.
